# Blockade of TGF-β/Smad signaling by the small compound HPH-15 ameliorates experimental skin fibrosis

**DOI:** 10.1186/s13075-018-1534-y

**Published:** 2018-03-15

**Authors:** Vu Huy Luong, Takenao Chino, Noritaka Oyama, Takashi Matsushita, Yoko Sasaki, Dai Ogura, Shin-ichiro Niwa, Tanima Biswas, Akiyuki Hamasaki, Mikako Fujita, Yoshinari Okamoto, Masami Otsuka, Hironobu Ihn, Minoru Hasegawa

**Affiliations:** 1 Department of Dermatology, Division of Medicine, Faculty of Medical Sciences, University of Fukui, Fukui, Japan; 2 Department of Dermatology, Faculty of Medicine, Institute of Medical, Pharmaceutical and Health Sciences, Kanazawa University, Kanazawa, Japan; 3 Link Genomics, Inc., Tokyo, Japan; 4 Department of Bioorganic Medicinal Chemistry, Kumamoto University, Kumamoto, Japan; 5 Research Institute for Drug Discovery, Kumamoto University, Kumamoto, Japan; 6 Department of Dermatology and Plastic Surgery, Faculty of Life Sciences, Kumamoto University, Kumamoto, Japan

**Keywords:** Systemic sclerosis, HPH-15, TGF-β, Macrophage, Fibroblast

## Abstract

**Background:**

Transforming growth factor-β (TGF-β)/Smad signaling is well known to play a critical role in the pathogenesis of systemic sclerosis (SSc). We previously developed an artificial molecule, the histidine-pyridine-histidine ligand derivative HPH-15, which may have an antifibrotic effect. The purpose of the present study was to clarify the effects of this drug in human skin fibroblasts and in a preclinical model of SSc.

**Methods:**

The effects of HPH-15 on expression of extracellular matrix components and TGF-β signaling in human dermal fibroblasts were analyzed. The antifibrotic properties of HPH-15 and its mechanisms were also examined in a bleomycin-induced skin fibrosis mouse model.

**Results:**

HPH-15 suppressed the TGF-β-induced phosphorylation of Smad3 and inhibited the expression of collagen I, fibronectin 1, connective tissue growth factor, and α-smooth muscle actin induced by TGF-β in cultured human skin fibroblasts. In the bleomycin-induced skin fibrosis model, oral administration of HPH-15 protected against the development of skin fibrosis and ameliorated established skin fibrosis. Additionally, HPH-15 suppressed the phosphorylation of Smad3 in various cells, including macrophages in the bleomycin-injected skin. Further, in the treated mice, dermal infiltration of proinflammatory macrophages (CD11b^+^Ly6C^hi^) and M2 profibrotic macrophages (CD11b^+^CD204^+^ or CD11b^+^CD206^+^) was significantly decreased during the early and late stages, respectively. HPH-15 treatment resulted in decreased messenger RNA (mRNA) expression of the M2 macrophage markers arginase 1 and *Ym-1* in the skin, whereas it inversely augmented expression of Friend leukemia integration 1 and Krüppel-like factor 5 mRNAs, the transcription factors that repress collagen synthesis. No apparent adverse effects of HPH-15 were found during the treatment.

**Conclusions:**

HPH-15 may inhibit skin fibrosis by inhibiting the phosphorylation of Smad3 in dermal fibroblasts and possibly in macrophages. Our results demonstrate several positive qualities of HPH-15, including oral bioavailability, a good safety profile, and therapeutic effectiveness. Thus, this TGF-β/Smad inhibitor is a potential candidate therapeutic for SSc clinical trials.

**Electronic supplementary material:**

The online version of this article (10.1186/s13075-018-1534-y) contains supplementary material, which is available to authorized users.

## Background

Systemic sclerosis (SSc), or scleroderma, is a systemic autoimmune disease of unknown etiology characterized by early inflammation and vascular injury followed by fibrosis of the skin and visceral organs [1, 2]. Excessive deposition of collagen and other extracellular matrix (ECM) components in the affected organs is the pathophysiological hallmark of SSc. Activated fibroblasts and α-smooth muscle actin (α-SMA)-positive myofibroblasts are largely responsible for excessive matrix synthesis and tissue deposition [3]. Fibroblast activation and myofibroblast transformation result from a complex series of events initiated by a number of profibrotic molecules, including transforming growth factor-β (TGF-β); connective tissue growth factor (CTGF); platelet-derived growth factor; interleukin (IL)-4, IL-6, and IL-13; and endothelin-1.

One set of the main inducers and producers of these mediators is macrophages. Mouse circulating monocytes can be divided into Ly6C^hi^ (classical or inflammatory monocytes) and Ly6C^lo^ (nonclassical or anti-inflammatory monocytes) [4]. After infiltrating the tissue, these monocytes can differentiate into macrophages in response to various stimuli dependent on the tissue microenvironment. The differentiated macrophages can be classified as classically activated inflammatory macrophages (M1) and alternatively activated tissue profibrotic macrophages (M2) [5]. M2 macrophages can induce and/or maintain tissue fibrosis by producing profibrotic cytokines, including IL-4, IL-13, and TGF-β. Among these, TGF-β is considered one of the most potent inducers of fibroblast activation and tissue fibrosis [6–8]. Binding of TGF-β to its cell surface receptors triggers intracellular signal transduction of Smad-dependent or Smad-independent pathways. In the Smad-dependent pathway, activation of TGF-β receptor type I leads to phosphorylation of Smad2 and Smad3, allowing these molecules to complex with Smad4 and translocate from the cytoplasm into the nucleus, where they bind to a consensus Smad-binding element within the 5′-flanking region of the targeted genes (or DNA). Upon binding to this element, activated Smad proteins recruit transcriptional cofactors to the targeted DNA, resulting in transcription of several key fibrotic genes, such as those encoding collagens and fibronectin [8, 9]. Sustained activation of TGF-β/Smad3 signaling has been detected in the skin fibroblasts of a bleomycin-induced SSc model, one of the most popular SSc models [10], and targeted disruption of this pathway inhibits bleomycin-induced skin fibrosis [11]. In addition, blockade of TGF-β signaling has been shown to reduce the development of skin fibrosis in several other experimental models [12–15]. However, molecular-based approaches targeting the TGF-β cascade have not been established for the treatment of patients with SSc. For this reason, we sought to identify novel therapeutic modalities for SSc. We previously reported a series of symmetrically substituted 2,6-pyridine derivatives, histidine-pyridine-histidine ligand (HPH), possessing varied biological activities dependent on the structure of the 2,6-substituents. These activities include antitumor activity [16, 17], inhibition of zinc finger proteins [18], and inhibition of nuclear factor-κB [19]. Among these, HPH-15, which was formerly named HPH-8, is an HPH derivative that has *S*-*tert*-butylcysteamine substituents (Additional file 1: Figure S1). We previously reported that HPH-15 has antiviral activities against herpes simplex virus 1, although the exact mechanism remains unknown [20]. In a preliminary study, we found novel antifibrotic activity of HPH-15 in several cell lines (Ogura D and Niwa S 2017, unpublished data).

In this study, we demonstrated that HPH-15 has antifibrotic effects in both a mouse model and cultured human dermal fibroblasts. Our findings indicate that HPH-15 inhibits fibrosis, at least partially, by antagonizing the phosphorylation of Smad3 in skin fibroblasts. Interestingly, HPH-15 reduces the infiltration of both inflammatory and profibrotic macrophages in bleomycin-injected skin.

## Methods

### Cell culture

Normal human dermal fibroblasts derived from neonatal foreskin were purchased (Kurabo Industries, Osaka, Japan) and grown in DMEM (Nacalai Tesque, Kyoto, Japan) containing 10% FBS, 100 U/ml penicillin, and 100 μg/ml streptomycin (Nacalai Tesque) at 37 °C in a humidified 5% CO_2_ atmosphere. When cells reached ~ 70% confluency, they were starved in DMEM containing 0.1% FBS for 24 h and then pretreated with 0.05% dimethyl sulfoxide (DMSO) as a control or various concentrations of DMSO-diluted HPH-15. One hour later, cells were stimulated with 10 ng/ml human recombinant TGF-β1 (PeproTech, Rocky Hill, NJ, USA) and were used for the indicated experiments. All experiments used fibroblasts of passages between 8 and 13.

### Animal studies

Female C57BL/6 mice ages 8–10 weeks (CLEA Japan, Tokyo, Japan) were used in two different animal trials for skin fibrosis according to a previous study with minor modification [21]. The preventive model (*n* = 4–6) was performed by daily subcutaneous injections of bleomycin (1 mg/ml in saline) into the shaved back of the mice (150 μl), concurrent with daily oral gavage of HPH-15 (100 mg/kg in sterilized olive oil) or vehicle alone (sterilized olive oil) for 1, 2, 3, or 4 weeks. Subcutaneous injections of 0.9% NaCl served as a control. In the curative model (*n* = 5), either bleomycin or saline was administered on alternate days for 6 weeks. Two weeks after the first injection, mice were given a daily dose of HPH-15 (100 mg/kg) or vehicle for the remaining 4 weeks. The HPH-15 doses were optimized on the basis of sequential pilot experiments (data not shown).

### Histologic analysis

Mouse skin was fixed in 10% formalin and then embedded in paraffin. Sections (6 μm in thickness) were subjected to H&E and Masson’s trichrome staining. For evaluation of skin fibrosis, dermal thickness was defined computationally as the thickness of the skin from the top of the granular layers to the junction between the dermis and subcutaneous fat [22] in five distinct fields at an equal magnification (×40) using a light microscope, and results were expressed as mean **±** SEM. Collagen deposition was quantified on Masson’s trichrome-stained sections as the ratio of blue-stained area to total stained area using Photoshop Elements version 12 software (Adobe Systems, San Jose, CA, USA).

### Sircol Soluble Collagen Assay

Collagen deposition and fibrosis were quantified as total soluble collagen using the Sircol Soluble Collagen Assay (Biocolor, Carrickfergus, UK). Briefly, full-thickness 4-mm punch biopsy samples of mouse back skin (*n* = 5–6) were homogenized in acid-pepsin solution (0.5 M acetic acid containing 1 mg/ml pepsin) for 48 h at 4 °C. After centrifugation, 1 ml of Sircol dye reagent was added to 100 μl of supernatant and incubated for 30 minutes. After the suspension was removed, droplets were dissolved in 1 ml of Sircol alkali reagent, and relative absorbance was measured at 555 nm.

### Western blot analysis

Total protein was extracted from human dermal fibroblasts using a total protein extraction kit (101Bio, Mountain View, CA, USA). Protein concentration was assessed using a spectrophotometer and a bicinchoninic acid protein assay kit (TaKaRa Bio, Shiga, Japan). Equal amounts of protein from each sample (40 μg) were subjected to SDS-PAGE on a Mini-PROTEAN TGX Precast gel and transferred to a nitrocellulose membrane (Bio-Rad Laboratories, Hercules, CA, USA). The blotted membrane was blocked for 30 minutes at room temperature with 5% skim milk/Tris-buffered saline with Tween 20 (TBS-T), followed by incubation with antiphosphorylated Smad3, anti-Smad3 (Cell Signaling Technology, Danvers, MA, USA), anti-collagen type I, alpha 2 (Col1a2; Abcam, Cambridge, UK), anti-fibronectin 1 (anti-FN1) (LSBio, Seattle, WA, USA), anti-CTGF (Santa Cruz Biotechnology, Dallas, TX, USA), or anti-glyceraldehyde 3-phosphate dehydrogenase (anti-GAPDH) (Thermo Fisher Scientific, Waltham, MA, USA) antibodies overnight at 4 °C. After being washed with TBS-T three times, the membrane was incubated for 1 h at room temperature with horseradish peroxidase-conjugated secondary antibody. The membrane was exposed to an enhanced chemiluminescence reagent, Chemi-Lumi One Super solution (Nacalai Tesque). Protein bands were quantified using ImageQuant TL software (version 7.0; GE Healthcare Life Sciences, Pittsburgh, PA, USA) and normalized against the loading control, GAPDH.

### Immunofluorescence staining of cultured mouse fibroblasts

After stimulation with recombinant TGF-β1 for 2 h (for detecting p-Smad3) or 24 h (for detecting α-SMA), cells were washed twice in ice-cold PBS. Then cells were fixed for 10 minutes at room temperature in 100% ethanol or 4% paraformaldehyde phosphate buffer for staining of p-Smad3 or α-SMA, respectively. Next, cells were permeabilized with 0.1% Triton X-100 in PBS for 3 minutes. Cells were blocked with 2% FBS for 30 minutes, incubated with anti-p-Smad3 antibody (1:50 in 2% FBS; Cell Signaling Technology) or anti-α-SMA antibody (1:300 in 2% FBS; Abcam) for 60 minutes at room temperature, and then with Alexa Fluor 488-conjugated goat antirabbit antibody for 40 minutes. Coverslips were mounted by using VECTASHIELD mounting medium with 4′,6-diamidino-2-phenylindole (DAPI) (Vector Laboratories, Burlingame, CA, USA).

### Immunohistochemical staining of mouse skin

Sections (6 μm in thickness) from paraffin-embedded mouse skin were incubated for 120 minutes at room temperature with monoclonal antibodies (mAbs) to CD3 (1:200; Nichirei Biosciences, Tokyo, Japan), F4/80 (1:1600; Abcam), and p-Smad3 (1:50; Santa Cruz Biotechnology), then with peroxidase-labeled secondary antibody (Nichirei Biosciences), followed by color development with the aminoethylcarbazole system (Nichirei Biosciences). CD3^+^ cells, F4/80^+^ cells, and p-Smad3-positive cells were counted under a high-power microscopic field (the Hall section for CD3^+^ cells and distinct fields for the F4/80^+^ cells and p-Smad3-positive cells). Each section was examined independently by two investigators (TC and NO) in a blinded manner.

### Immunofluorescence staining of mouse skin

The 4-μm cryosections from bleomycin-injected mouse skin were incubated at 4 °C overnight with the primary antibodies p-Smad3 (1:50; Santa Cruz Biotechnology) and F4/80 (1:200; Abcam). Antibodies conjugated with Alexa Fluor 488 or Alexa Fluor 594 (Thermo Fisher Scientific) were used as secondary antibodies of p-Smad3 or F4/80, respectively. Coverslips were mounted by using VECTASHIELD mounting medium with DAPI (Vector Laboratories).

### Preparation of skin cell suspension

A 2 × 2.5-cm piece of depilated back skin was minced and then digested in 7 ml of RPMI 1640 medium containing 10% FBS and 2 mg/ml crude collagenase (Sigma-Aldrich, St. Louis, MO, USA), 1.5 mg/ml hyaluronidase (Sigma-Aldrich), and 0.03 mg/ml DNase I (Roche Applied Science, Indianapolis, IN, USA) at 37 °C for 90 minutes [23]. Samples were passed through a 70-μm Falcon cell strainer (Fisher Scientific/BD Biosciences, Pittsburgh, PA, USA) to obtain single-cell suspensions. After centrifugation at 1500 rpm for 5 minutes, the cell pellet was resuspended in a 70% Percoll solution (GE Healthcare Life Sciences) and then overlaid with a 37% Percoll solution, followed by centrifugation at 1800 rpm for 20 minutes. The cells were aspirated from the Percoll interface and passed through a 70-μm Falcon cell strainer. The harvested cells were washed with ice-cold PBS and used for flow cytometric analysis.

### Flow cytometry

mAbs against the following mouse antigens were used: Alexa Fluor 488-conjugated anti-CD45, allophycocyanin (APC)-conjugated anti-Ly6G, Pacific Blue-conjugated anti-CD11b, peridinin-chlorophyll-conjugated anti-Ly6C, phycoerythrin-cyanine 7-conjugated anti-CD206 (all from BioLegend, San Diego, CA, USA), and APC-conjugated anti-CD204 (R&D Systems, Minneapolis, MN, USA). To distinguish dead cells from live cells, the LIVE/DEAD Fixable Aqua Dead Cell Stain Kit (Thermo Fisher Scientific) was used.

The single-cell suspensions obtained as described above were stained for 20 minutes at 4 °C using the indicated mAbs at predetermined optimal concentrations for six-color immunofluorescence analysis. Stained samples were analyzed using the FACSCanto II system (BD Biosciences, San Jose, CA, USA). Data were analyzed using FlowJo software version 7 (FlowJo, Ashland, OR, USA).

### RT-PCR

Total RNA was isolated from the skin or cultured fibroblasts using RNeasy spin columns (Qiagen, Valencia, CA, USA) and digested with DNase I (Qiagen) to remove chromosomal DNA. Total RNA was reverse-transcribed to a complementary DNA using a reverse transcription system with random hexamers (TaKaRa Bio). Real-time RT-PCR was performed using the StepOnePlus Real-Time PCR System (Thermo Fisher Scientific). All data were normalized against *GAPDH* messenger RNA (mRNA) and quantified as relative expression.

### Statistical analysis

All data are shown as the mean ± SEM and were analyzed using Prism software version 7 (GraphPad Software, La Jolla, CA, USA). The significance of differences between samples was determined by Student’s two-tailed *t* test. *p* Values ≤ 0.05 were considered statistically significant.

## Results

### HPH-15 inhibits TGF-β-induced fibrotic responses in cultured human dermal fibroblasts

**Fig. 1:**
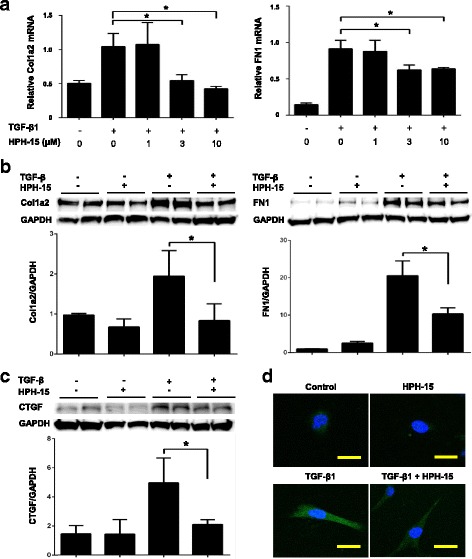
The antifibrotic role of the histidine-pyridine-histidine ligand derivative HPH-15 in cultured human dermal fibroblasts stimulated with transforming growth factor-β1 (TGF-β1). Normal human dermal fibroblasts were pretreated with dimethyl sulfoxide (DMSO) or various concentrations of DMSO-diluted HPH-15 for 1 h. Then, cells were stimulated with 10 ng/ml human TGF-β1 for the next 24 h. **a** Messenger RNA (mRNA) expression of collagen type I, alpha 2 (*Col1a2*), and fibronectin 1 (*FN1*) in fibroblasts was measured by real-time RT-PCR. *n* = 3 per group. **b–d** HPH-15 at a dose of 3 μM was used on the basis of results shown in (**a**). **b** and **c** Total cell lysates were subjected to Western blotting of Col1a2, FN1, and connective tissue growth factor (CTGF). *n* = 4 per group. Values are normalized relative to control. **d** Normal human fibroblasts were stained with α-smooth muscle actin by immunofluorescent staining, and representative pictures are shown. Scale bar = 30 μm. *n* = 3 per group. All values represent mean ± SEM. * *p* ≤ 0.05. *GAPDH* Glyceraldehyde 3-phosphate dehydrogenase

Fibroblasts are the main source of collagen and other ECM components in fibrotic skin. We first examined the biological effects of HPH-15 on in vitro ECM synthesis (relevant to the fibroblastic activity) using human dermal fibroblasts. As shown in Fig. 1a, recombinant TGF-β1 stimulated baseline expression of *Col1a2* and *FN1* mRNAs by 24 h of treatment. A 1-h pretreatment with 3 or 10 μM of HPH-15 significantly suppressed the TGF-β1-dependent induction of both mRNAs, although 1 μM HPH-15 had no effect. The suppression reached a sustained maximal level with 3 μM HPH-15. Moreover, the TGF-β1-dependent increase of *Col1a2* mRNA was decreased almost to the steady-state level at this dose.

Therefore, we confirmed the effect of 3 μM HPH-15 for protein expression of Col1a2, FN1, and CTGF, a representative TGF-β1-responsive profibrotic mediator, using Western blotting (Fig. 1b and c). TGF-β1-induced protein expression of Col1a2, FN1, and CTGF on human dermal fibroblasts was significantly inhibited by HPH-15 treatment. Additionally, immunofluorescent cell staining demonstrated that preincubation with HPH-15 inhibited the TGF-β1-induced α-SMA expression on dermal fibroblasts (Fig. 1d). The administration of HPH-15 did not significantly affect the expression of these proteins in the unstimulated fibroblasts. These data suggest that pretreatment with HPH-15 efficiently inhibits the fibrogenic activity induced by TGF-β1 in dermal fibroblasts.

### HPH-15 antagonizes phosphorylation of Smad3 in cultured human dermal fibroblasts

The Smad signaling pathways mediate the transcriptional regulation of genes induced by TGF-β superfamily members. To investigate the antifibrotic effects of HPH-15 on the Smad-dependent pathway, we evaluated the expression of p-Smad3 in TGF-β1-treated human dermal fibroblasts.

**Fig. 2:**
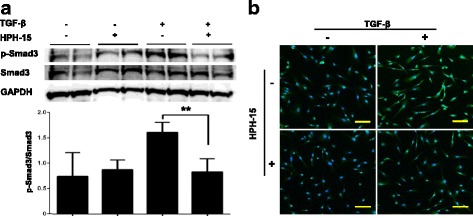
The histidine-pyridine-histidine ligand derivative HPH-15 antagonizes the phosphorylation of Smad3 in cultured human dermal fibroblasts. **a** and **b** Normal human fibroblasts were pretreated with dimethyl sulfoxide (DMSO) or DMSO-diluted HPH-15 (3 μM) for 1 h. Then, cells were stimulated with 10 ng/ml human transforming growth factor-β1 (TGF-β1) for the next 3 h. **a** Phosphorylation of Smad3 in the cultured human dermal fibroblasts was assessed by Western blot analysis. *n* = 4 per group. Values are normalized relative to control. All values represent mean ± SEM. ** *p* ≤ 0.01. **b** Human fibroblasts were immunostained for p-Smad3 (*green*). Nuclei (*blue*) was stained with 4′,6-diamidino-2-phenylindole. Scale bar = 100 μm. *n* = 3 per group. *GAPDH* Glyceraldehyde 3-phosphate dehydrogenase

Western blot assays demonstrated that HPH-15 significantly attenuates the TGF-β1-induced phosphorylation of Smad3 without affecting total Smad expression in cultured human fibroblasts (Fig. 2a). Immunocytochemistry revealed that preincubation with HPH-15 efficiently abrogated the TGF-β1-dependent increase of Smad3 phosphorylation in cultured human dermal fibroblasts (Fig. 2b). HPH-15 did not significantly affect the phosphorylation of Smad3 in human dermal fibroblasts not stimulated with TGF-β1.

### HPH-15 ameliorates bleomycin-induced skin fibrosis in mice

**Fig. 3:**
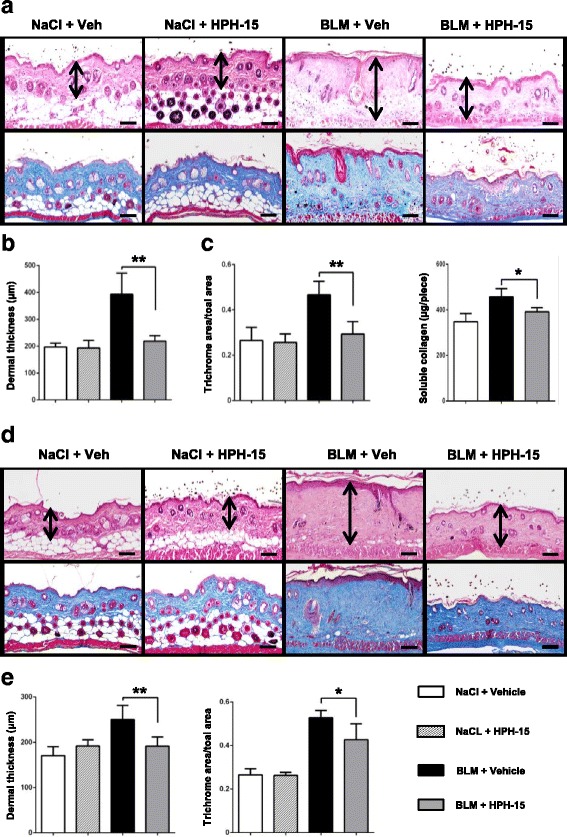
Histidine-pyridine-histidine ligand derivative HPH-15 treatment ameliorates bleomycin (BLM)-induced skin fibrosis in mice. The antifibrotic effects of HPH-15 were analyzed at the endpoint of the preventive model (**a–c**) and curative model (**d** and **e**). The preventive model was performed by daily subcutaneous injections of BLM into the back skin of the mice concurrently with daily oral gavage of HPH-15 or vehicle (sterilized olive oil) for 4 weeks. In the curative model, BLM was administered on alternate days for 6 weeks. Two weeks after the first injection, mice were given a daily dose of HPH-15 or vehicle for the remaining 4 weeks. Representative images of H&E-stained (*upper pictures*) and Masson’s trichrome-stained (*lower pictures*) tissue. Arrows indicate dermal thickness. Scale bar = 100 μm. Skin fibrosis was compared by determining dermal thickness, ratio of trichrome area/total area, and soluble collagen content. All values represent mean ± SEM; *n* = 4–6 per group. * *p* ≤ 0.05; ** *p* ≤ 0.01

Using a bleomycin-induced skin fibrosis mouse model, we attempted to examine the in vivo antifibrotic effects of HPH-15 at two different treatment onsets, one in a preventive model (ongoing) and the other in a curative model (postonset). In the preventive experiment, the local bleomycin injection and oral HPH-15 were coadministered daily for 4 weeks. Histology of the bleomycin-injected skin sites revealed a marked decrease in the dermal thickness (89% decrease) and collagen deposition compared with mice coadministered sterilized olive oil as a control (Fig. 3a and b). In addition, the soluble collagen fraction in the bleomycin-injected skin was significantly reduced following the coadministration of HPH-15 (Fig. 3c). HPH-15 treatment did not affect the dermal thickness in the saline-injected skin (Fig. 3a and b). These data suggest that the coadministration of HPH-15 can counteract the fibrogenic activity of bleomycin in vivo.

To evaluate the effects of HPH-15 on established fibrosis, bleomycin or a saline control was administered to mice every other day. Two weeks after the first injection of bleomycin, significant skin fibrosis was detected (Additional file 2: Figure S2). At this point, mice were given a daily dose of HPH-15 or vehicle control for the remaining 4 weeks while the bleomycin injection was continued on alternate days. Treatment with HPH-15 resulted in significantly decreased dermal thickness (71% decrease) in the lesional skin compared with the vehicle-treated mice. Moreover, the Masson’s trichrome-stained area was significantly reduced in the HPH-15-treated mice (Fig. 3d and e). HPH-15 was well tolerated throughout all experiments, and no significant effects on body weight loss (Additional file 3: Figure S3) or other signs of toxicity were noted.

### HPH-15 inhibits inflammatory cell infiltration during early stage of bleomycin injection

**Fig. 4:**
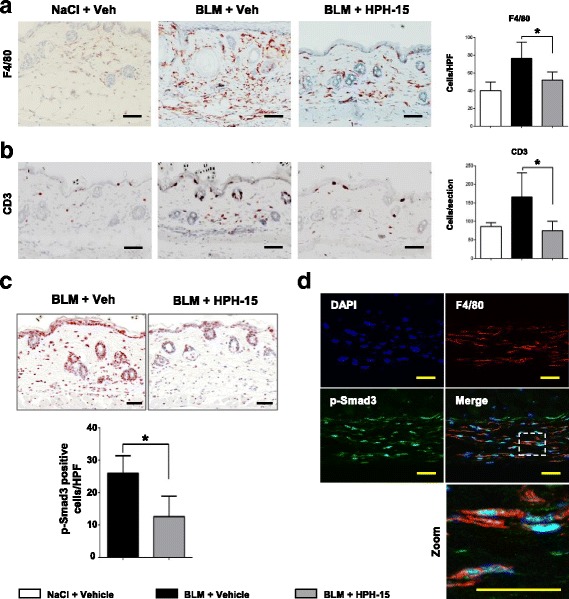
The histidine-pyridine-histidine ligand derivative HPH-15 inhibits leukocyte infiltration and suppresses Smad3 phosphorylation in bleomycin (BLM)-injected skin. **a–d** Back skin samples of mice treated with HPH-15 or vehicle were harvested after 14 days of daily BLM injections. Skin sections were incubated with anti-F4/80 antibody (**a**) and anti-CD3 antibody (**b**). Representative images are shown on the *left*. Scale bar = 50 μm. Quantitative analysis is shown in the bar graph on the *right*. **c** Skin sections were stained for p-Smad3. Scale bar = 50 μm. Quantitative analysis of p-Smad3-positive cells in the dermis is shown in the bar graph. **d** Cryosections of the skin were subjected to immunofluorescent staining for 4′,6-diamidino-2-phenylindole (DAPI) (nuclear, *blue*), p-Smad3 (nuclear, *green*), and F4/80 (cell surface, *red*). Dashed box inset indicates the location of zoomed image. Scale bar = 30 μm. All values represent mean ± SEM; *n* = 5 per group. * *p* ≤ 0.05. *HPF* High-power field

It is known that injection of bleomycin induces an early and transient inflammatory response in the dermis. Among the infiltrating cells, macrophages are the most prominent [10]. Consistent with this, local injection of bleomycin, but not control saline, induced the infiltration of F4/80-positive macrophages into the middle dermis at day 14 (Fig. 4a). This increased infiltration of macrophages was significantly blocked by concurrent administration of HPH-15 (*p* < 0.05). Similar inhibitory effects of HPH-15 were observed on the local infiltration of CD3-positive T lymphocytes in bleomycin-treated skin (Fig. 4b). These results show the in vivo preventive effects of HPH-15 during the early inflammatory response of bleomycin-induced skin fibrosis.

### HPH-15 suppresses phosphorylation of Smad3 in various cells, including macrophages, in bleomycin-injected skin

In bleomycin-injected mouse skin, immunohistochemical staining revealed a downregulated expression of p-Smad3 in the epidermis, dermis, and subcutaneous tissues of HPH-15-treated mice. The number of p-Smad3-positive cells in the dermis and subcutaneous tissues (suggestive of fibroblasts and leukocytes) was significantly decreased in the HPH-15-treated mice compared with control mice (Fig. 4c). In addition, immunofluorescence staining indicated that most infiltrating p-Smad3-positive cells were F4/80-positive macrophages in the bleomycin-injected skin (Fig. 4d).

### HPH-15 reduces infiltration of inflammatory CD11b^+^Ly6C^hi^ monocytes during early-stage fibrosis and profibrotic M2 macrophages during late-stage fibrosis

**Fig. 5:**
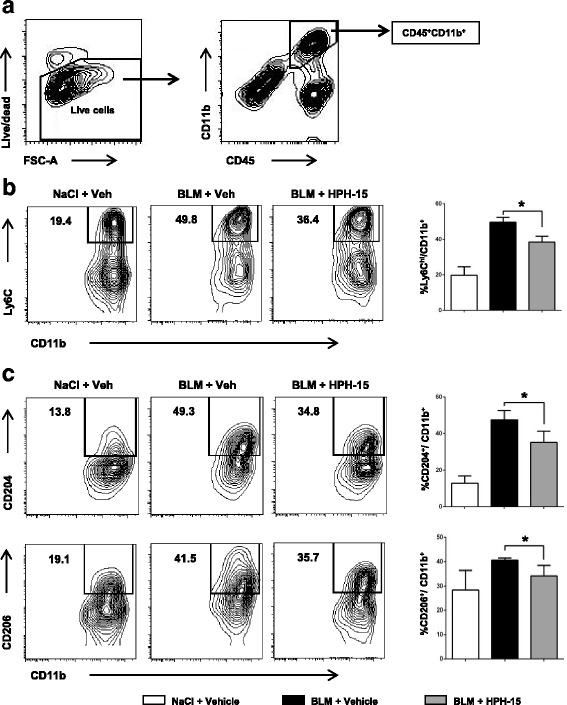
The histidine-pyridine-histidine ligand derivative HPH-15 reduces the infiltration of inflammatory CD11b^+^Ly6C^hi^ monocytes in the early stage of fibrosis and profibrotic M2 macrophages during the late stage. Skin samples from normal, vehicle-treated, and HPH-15-treated mice were harvested 7 or 21 days after bleomycin (BLM) injection. **a** CD45^+^CD11b^+^ cells were gated for analysis of the subsets of macrophages. Representative images (*left*) and bar graphs (*right*) of Ly6C^hi^ macrophages on day 7 (**b**) and CD204^+^ and CD206^+^ M2 macrophages on day 21 (**c**) analyzed by flow cytometry. All values represent mean ± SEM; *n* = 3 per group. * *p* ≤ 0.05. *FSC-A* Forward scatter area

As described above, the main infiltrating cell population in bleomycin-injected skin is macrophages (Fig. 4). To further investigate the effects of HPH-15 on subsets of macrophages, we isolated leukocytes from lesional skin at two different time points—the inflammatory stage (day 7) and the fibrotic stage (day 21)—and stained for monocyte/macrophage surface markers. The infiltration of CD11b^+^Ly6C^hi^ monocytes was remarkably increased after 7 days of bleomycin injections. Moreover, HPH-15 treatment significantly decreased the percentage of CD11b^+^Ly6C^hi^ monocytes in the skin (Fig. 5b).

**Fig. 6:**
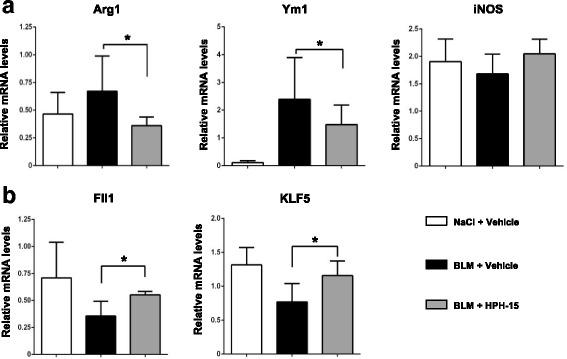
The effect of the histidine-pyridine-histidine ligand HPH-15 on the messenger RNA (mRNA) expression of macrophage markers and transcription factors. Skin samples from normal, vehicle-treated, and HPH-15-treated mice were harvested 14 days after bleomycin (BLM) injection. Total RNA was extracted, and expression of arginase 1 (*Arg1*), *Ym1*, inducible NO synthase (*iNOS*) (**a**), Friend leukemia integration 1 (*Fli1*), and Krüppel-like factor 5 (*KLF5*) (**b**) mRNAs were quantitatively analyzed by real-time RT-PCR. Each sample was repeated in duplicate. All values represent mean ± SEM; *n* = 4–5 per group. * *p* ≤ 0.05

M2 macrophages are well known to play a central role in the pathologic fibrotic response [24]. Therefore, we next evaluated the impact of HPH-15 on the M2 macrophage population using the M2 macrophage cell surface markers CD204 and CD206. On day 21, mice treated with bleomycin exhibited an increase in the proportion of CD11b^+^CD204^+^ and CD11b^+^CD206^+^ cells in the skin. HPH-15 administration significantly reduced these subsets (Fig. 5c) as well as the expression of *Arg1* and *Ym1* mRNAs, established markers of M2 macrophages, in the skin lesions of bleomycin-treated mice (Fig. 6a).

### HPH-15 restores mRNA expression of Fli1 and KLF5 in bleomycin-injected skin

Friend leukemia integration 1 (Fli1) and Krüppel-like factor 5 (KLF5) are transcription factors that repress expression of the collagen gene [25, 26]. Researchers in previous studies reported decreased mRNA levels of these transcription factors in murine skin fibrosis models [27]. We found that bleomycin injection caused remarkably decreased mRNA expression of *Fli1* and *KLF5*, and, as we expected, expression of *Fli1* and *KLF5* mRNAs were significantly recovered by HPH-15 treatment of bleomycin-injected mice (Fig. 6b).

## Discussion

We developed an orally active agent, HPH-15, and investigated its effects on fibrosis both in vivo and in vitro. HPH-15 disrupted the phosphorylation of Smad3 in human skin fibroblasts stimulated with TGF-β and effectively inhibited the expression of α-SMA, Col1a2, FN1, and CTGF. Furthermore, HPH-15 not only prevented skin fibrosis but also was effective against established skin fibrosis in the bleomycin-induced model. Treatment with HPH-15 inhibited the phosphorylation of Smad3 in keratinocytes, fibroblasts, and leukocytes (most of them being macrophages) in fibrotic skin and reduced the accumulation of inflammatory macrophages and profibrotic M2 macrophages in the early and late phases, respectively.

We previously synthesized a series of pyridine-based symmetrical molecules. Among these, HPH-15 showed promise as an antifibrotic agent in our preliminary cellular experiments (Ogura D and Niwa S 2017, unpublished data). In the present study, we confirmed that HPH-15 blocks Smad3 phosphorylation in human skin fibroblasts stimulated with TGF-β1. Furthermore, HPH-15 suppresses the TGF-β1-dependent expression of α-SMA, ECM components, and CTGF, a representative TGF-β1-responsive mediator, on human skin fibroblasts. These findings suggest that HPH-15 inhibits skin fibrosis via suppression of TGF-β/Smad signaling of fibroblasts. Recently, fresolimumab, a human immunoglobulin G4κ mAb that neutralizes all three TGF-β isoforms, was used in an open-label trial in patients with early diffuse SSc [28]. Interestingly, fresolimumab exhibited a tendency to decrease expression of macrophage/monocyte-associated genes in the skin. In our mouse experiments, macrophage accumulation was inhibited by HPH-15 in a manner similar to that of the antihuman TGF-β mAb. Although TGF-β is well known for its anti-inflammatory functions, it can also directly induce monocyte migration in vitro [29]. Furthermore, recombinant TGF-β injection induces macrophage infiltration in addition to fibrosis in mouse skin [30]. Although mice with the stiff skin syndrome mutation, a genetic mouse model of SSc, show the presence of increased proinflammatory cells, including plasmacytoid dendritic cells, T-helper cells, and plasma cells in the skin, TGF-β-neutralizing antibody inhibited the inflammation and reversed the established skin fibrosis [31]. Therefore, blockade of TGF-β signaling may directly inhibit the early inflammation that leads to fibrosis.

The presence of immunocyte infiltrate is usually detected in the skin of patients with early SSc [32]. Similar inflammatory cell infiltration is also found in the skin of the bleomycin-induced skin fibrosis model [33, 34]. Macrophages are an important source of fibrotic cytokines, including TGF-β, and have been considered to play a central role in the pathogenesis of fibrotic disorders, including SSc [35, 36]. Specifically, M2 macrophages have been implicated as critical mediators in various tissue fibrosis models, including bleomycin-induced skin fibrosis [5, 24, 37]. Importantly, gene expression analysis demonstrated augmentation of the M2 macrophage signature in the skin of patients with SSc, which was suppressed by anti-IL-6R mAb (tocilizumab) therapy associated with an improvement of fibrosis [38]. In bleomycin-injected skin, the majority of immune cells were macrophages, and HPH-15 markedly inhibited this accumulation. In addition, most p-Smad3-positive infiltrating cells were F4/80-positive macrophages in the bleomycin-injected skin, and those cells were dramatically decreased by HPH-15 treatment. Therefore, HPH-15 may directly affect macrophages in addition to fibroblasts in bleomycin-injected fibrotic skin.

Circulating monocytes can be classified into Ly6C^hi^ and Ly6C^low^ monocytes [4]. Ly6C^hi^ or inflammatory monocytes respond to inflammatory signals and leave the circulation by extravasation, whereas Ly6C^low^ monocytes patrol the luminal side of the vasculature [39, 40]. Recent studies demonstrated that Ly6C^hi^ inflammatory monocytes are increased in the tissues of mouse models, including lung fibrosis and unilateral ureteral obstruction-induced fibrosis, during the progressive fibrotic phase [41, 42]. Depending on the specific cytokines to which they are exposed, tissue Ly6C^hi^ macrophages will polarize into inflammatory macrophages or downregulate Ly6C and polarize cells into tissue-remodeling/profibrotic (M2) macrophages [4, 39, 43]. In our experiments, following 7 days of bleomycin injections, there was a remarkable increase in the Ly6C^hi^ macrophage population in the skin, an effect that was significantly inhibited by administration of HPH-15. During the fibrotic stage (day 21), HPH-15 treatment inhibited the expansion of the M2 macrophage population and specifically caused a reduction of the CD11b^+^CD204^+^ and CD11b^+^CD206^+^ subsets. Although it would be ideal to compare numbers of infiltrating Ly6C^hi^ and M2 macrophages rather than just their frequencies, this proved to be technically difficult owing to the challenge of cell isolation from the skin. However, considering the immunohistochemical findings regarding total macrophages, the effect of HPH-15 on the reduction of these macrophage subsets is likely dramatic. Furthermore, HPH-15 inhibited expression of arginase 1 and *Ym1* mRNAs, markers of M2 macrophages, in bleomycin-treated skin. M2 macrophages likely appear during the fibrotic stage, either via differentiation of newly recruited infiltrating macrophages or by in situ transition of previously differentiated infiltrating M1 macrophages or Ly6C^hi^ inflammatory macrophages in the presence of Th2 cytokines such as IL-4 [24, 42, 44]. Additionally, a recent study demonstrated that TGF-β skews macrophage polarization toward an M2-like phenotype in human THP-1 macrophages [45]. Interestingly, TGF-β1/Smad3 signaling was critical for the transition of bone marrow-derived macrophages into collagen-producing myofibroblasts in a renal fibrosis mouse model of unilateral ureteric obstruction [46]. In that study, the macrophage myofibroblast transition was induced more predominantly in M2 macrophages.

In our study, the prevalence of Ly6C^hi^ monocytes was remarkably decreased in the skin of wild-type mice following bleomycin injection (from 49.6% on day 7 to 21.3% on day 21). Inversely, CD206^+^ M2 macrophages increased from 18.2% on day 7 to 40.5% on day 21 (Additional file 4: Figure S4). Although the detailed mechanisms regarding how HPH-15 reduces M2 macrophages in the skin remain unknown, the reduction of Ly6C^hi^ inflammatory macrophages during the inflammatory stage by HPH-15 treatment may partially explain this effect. In addition, inhibition of TGF-β function by HPH-15 might attenuate M2-polarized differentiation. Further studies are needed to confirm whether HPH-15 or the blockade of TGF-β/Smad signaling via other mechanisms inhibits the differentiation of Ly6C^hi^ macrophages into M2 macrophages.

Fli1 and KLF5 are transcription factors that repress collagen gene expression. The expression of both Fli1 and KLF5 were found to be decreased in SSc dermal fibroblasts [27, 47]. Importantly, heterozygous deficiency of both Fli1 and KLF5 results in the development of all three features of SSc, including autoimmunity, vasculopathy, and fibrosis [27]. More severe skin fibrosis and increased numbers of M2 macrophages were detected in Fli1-haploinsufficient mice by bleomycin injection [48]. Interestingly, Fli1 deficiency in epithelial cells spontaneously induces the fibrosis of skin, esophagus, and lungs as well as autoimmunity [49]. Consistent with these recent findings, our study demonstrates that *Fli1* and *KLF5* are downregulated in bleomycin-injected skin compared with normal skin. Moreover, these transcription factors were recovered with a concomitant amelioration of skin fibrosis in HPH-15-treated mice.

TGF-β has been considered to play a pivotal role in initiating and sustaining the fibrotic process in SSc, a function that is mediated via both canonical (Smad-dependent) and noncanonical (Smad-independent) pathways [2]. Fibroblasts from patients with SSc show constitutive Smad2/3 phosphorylation and nuclear localization, and various levels of abnormal Smad signaling have been detected. Therefore, targeting TGF-β/Smad signaling is an attractive strategy for treatment of SSc. Our data in vivo and in vitro showed that HPH-15 inhibits the phosphorylation of Smad3 protein in human dermal fibroblasts. The inhibition of Smad3 phosphorylation, thereby interfering with its binding to Smad-binding elements, likely results in the reduction of transcription of fibrotic genes such as *Col1a2* and *FN1*, as seen in cultured fibroblasts. Additionally, our limited data suggest that HPH-15 suppresses the Smad3 phosphorylation of dermal macrophages in bleomycin-injected skin.

Several points remain unclear, and future studies are required to address these. First, the effective concentration of HPH-15 should be improved if possible, such as by application of a drug delivery system or through structural optimization of HPH-15 before a clinical trial is conducted. Second, the effect of HPH-15 was investigated in only one mouse model of SSc. It has been suggested that the antifibrotic effects of specific molecules for SSc trials should be confirmed in at least two complementary animal models of SSc [50]. Therefore, further studies using other SSc models are required to clarify the utility of HPH-15. Third, the detailed mechanism of action of HPH-15, especially for macrophages and keratinocytes, should be determined in addition to fibroblasts.

## Conclusions

Our study shows that HPH-15 inhibits TGF-β/Smad signaling and fibrogenic activity of human skin fibroblasts in vitro and attenuates skin inflammation and subsequent fibrosis in a mouse model of SSc. Because the findings of this early preclinical study demonstrate several advantages of HPH-15, including the fact that it is an orally active agent with an excellent safety profile, HPH-15 shows potential as a candidate for SSc clinical trials.

## Additional files

Additional file 1: **Figure S1.** The molecular structure of HPH-15. (PDF 7 kb)

Additional file 2: **Figure S2**. HPH-15 treatment did not affect the growing of the mice. The body weight changes of mice treated with vehicle or HPH-15 were assessed every 3 days. All values represent mean ± SEM; *n* = 5 in each group. (PDF 6 kb)

Additional file 3: **Figure S3**. Every-other-day injection of bleomycin for 2 weeks induced significant skin fibrosis. Back skin of NaCl- or bleomycin-injected mice was harvested on day 14 and stained with H&E. Scale bar = 100 μm. *n* = 3 in each group. (PDF 80 kb)

Additional file 4: **Figure S4.** The inverse proportional change of Ly6C^hi^ macrophages and CD206^+^ M2 macrophages from the inflammation stage (day 7) to the fibrotic stage (day 21). The single-cell suspension obtained from the back skin of bleomycin-injected C57BL/6 mice on day 7 and day 21 was stained with the mAbs against CD45, CD11b, Ly6C, and CD206. Stained samples were analyzed using the FACSCanto II system. Data were analyzed using FlowJo software version 7. All values represent mean ± SEM. *n* = 3 at each time point. ** *p* ≤ 0.01. (PDF 49 kb)

## Abbreviations

APC: Allophycocyanin
Arg1: Arginase 1
BLM: Bleomycin
Col1a2: Collagen type I, alpha 2
CTGF: Connective tissue growth factor
DAPI: 4′,6-Diamidino-2-phenylindole
DMSO: Dimethyl sulfoxide
ECM: Extracellular matrix
Fli1: Friend leukemia integration 1
FN1: Fibronectin 1
FSC-A: Forward scatter area
GAPDH: Glyceraldehyde 3-phosphate dehydrogenase
HPF: High-power field
HPH: Histidine-pyridine-histidine ligand
IL: Interleukin
iNOS: Inducible NO synthase
KLF5: Krüppel-like factor 5
mAb: Monoclonal antibody
mRNA: Messenger RNA
SSc: Systemic sclerosis
TBS-T: Tris-buffered saline with Tween 20
TGF-β: Transforming growth factor-β
α-SMA: α-Smooth muscle actin

## References

[1] Allanore Y, Simms R, Distler O, Trojanowska M, Pope J, Denton CP, Varga J (2015). Systemic sclerosis. Nat Rev Dis Primers..

[2] Bhattacharyya S, Wei J, Varga J (2011). Understanding fibrosis in systemic sclerosis: shifting paradigms, emerging opportunities. Nat Rev Rheumatol..

[3] Rosenbloom J, Castro SV, Jimenez SA (2010). Narrative review: fibrotic diseases: cellular and molecular mechanisms and novel therapies. Ann Intern Med..

[4] Yona S, Jung S (2010). Monocytes: subsets, origins, fates and functions. Curr Opin Hematol..

[5] Mosser DM, Edwards JP (2008). Exploring the full spectrum of macrophage activation. Nat Rev Immunol..

[6] Roberts AB, Heine UI, Flanders KC, Sporn MB (1990). Transforming growth factor-β: major role in regulation of extracellular matrix. Ann N Y Acad Sci..

[7] Lafyatis R (2014). Transforming growth factor β at the centre of systemic sclerosis. Nat Rev Rheumatol..

[8] Liu Q, Chu H, Ma Y, Wu T, Qian F, Ren X, Tu W, Zhou X, Jin L, Wu W (2016). Salvianolic acid B attenuates experimental pulmonary fibrosis through inhibition of the TGF-β signaling pathway. Sci Rep..

[9] Feng XH, Derynck R (2005). Specificity and versatility in TGF-β signaling through Smads. Annu Rev Cell Dev Biol..

[10] Takagawa S, Lakos G, Mori Y, Yamamoto T, Nishioka K, Varga J (2003). Sustained activation of fibroblast transforming growth factor-β/Smad signaling in a murine model of scleroderma. J Invest Dermatol..

[11] Lakos G, Takagawa S, Chen SJ, Ferreira AM, Han G, Masuda K, Wang XJ, DiPietro LA, Varga J (2004). Targeted disruption of TGF-β/Smad3 signaling modulates skin fibrosis in a mouse model of scleroderma. Am J Pathol..

[12] Hasegawa M, Matsushita Y, Horikawa M, Higashi K, Tomigahara Y, Kaneko H, Shirasaki F, Fujimoto M, Takehara K, Sato S (2009). A novel inhibitor of Smad-dependent transcriptional activation suppresses tissue fibrosis in mouse models of systemic sclerosis. Arthritis Rheum..

[13] Distler JH, Jungel A, Huber LC, Schulze-Horsel U, Zwerina J, Gay RE, Michel BA, Hauser T, Schett G, Gay S (2007). Imatinib mesylate reduces production of extracellular matrix and prevents development of experimental dermal fibrosis. Arthritis Rheum..

[14] Wei J, Zhu H, Komura K, Lord G, Tomcik M, Wang W, Doniparthi S, Tamaki Z, Hinchcliff M, Distler JH (2014). A synthetic PPAR-γ agonist triterpenoid ameliorates experimental fibrosis: PPAR-γ-independent suppression of fibrotic responses. Ann Rheum Dis..

[15] Ruzehaji N, Frantz C, Ponsoye M, Avouac J, Pezet S, Guilbert T, Luccarini JM, Broqua P, Junien JL, Allanore Y (2016). Pan PPAR agonist IVA337 is effective in prevention and treatment of experimental skin fibrosis. Ann Rheum Dis..

[16] Ali TF, Iwamaru K, Ciftci HI, Koga R, Matsumoto M, Oba Y, Kurosaki H, Fujita M, Okamoto Y, Umezawa K (2015). Novel metal chelating molecules with anticancer activity: striking effect of the imidazole substitution of the histidine-pyridine-histidine system. Bioorg Med Chem..

[17] Hamasaki A, Naka H, Tamanoi F, Umezawa K, Otsuka M (2003). A novel metal-chelating inhibitor of protein farnesyltransferase. Bioorg Med Chem Lett..

[18] Radwan MO, Sonoda S, Ejima T, Tanaka A, Koga R, Okamoto Y, Fujita M, Otsuka M (2016). Zinc-mediated binding of a low-molecular-weight stabilizer of the host anti-viral factor apolipoprotein B mRNA-editing enzyme, catalytic polypeptide-like 3G. Bioorg Med Chem..

[19] Kanemaru Y, Momiki Y, Matsuura S, Horikawa T, Gohda J, Inoue J, Okamoto Y, Fujita M, Otsuka M (2011). An artificial copper complex incorporating a cell-penetrating peptide inhibits nuclear factor-k B (NF-κB) activation. Chem Pharm Bull..

[20] Hosono T, Yokomizo K, Hamasaki A, Okamoto Y, Okawara T, Otsuka M, Mukai R, Suzuki K (2008). Antiviral activities against herpes simplex virus type 1 by HPH derivatives and their structure-activity relationships. Bioorg Med Chem Lett..

[21] Huang J, Beyer C, Palumbo-Zerr K, Zhang Y, Ramming A, Distler A, Gelse K, Distler O, Schett G, Wollin L (2016). Nintedanib inhibits fibroblast activation and ameliorates fibrosis in preclinical models of systemic sclerosis. Ann Rheum Dis..

[22] Tanaka C, Fujimoto M, Hamaguchi Y, Sato S, Takehara K, Hasegawa M (2010). Inducible costimulator ligand regulates bleomycin-induced lung and skin fibrosis in a mouse model independently of the inducible costimulator/inducible costimulator ligand pathway. Arthritis Rheum..

[23] Huu DL, Matsushita T, Jin G, Hamaguchi Y, Hasegawa M, Takehara K, Fujimoto M (2013). FTY720 ameliorates murine sclerodermatous chronic graft-versus-host disease by promoting expansion of splenic regulatory cells and inhibiting immune cell infiltration into skin. Arthritis Rheum..

[24] Wermuth PJ, Jimenez SA (2015). The significance of macrophage polarization subtypes for animal models of tissue fibrosis and human fibrotic diseases. Clin Transl Med..

[25] Asano Y, Bujor AM, Trojanowska M (2010). The impact of Fli1 deficiency on the pathogenesis of systemic sclerosis. J Dermatol Sci..

[26] Dong JT, Chen C (2009). Essential role of KLF5 transcription factor in cell proliferation and differentiation and its implications for human diseases. Cell Mol Life Sci..

[27] Noda S, Asano Y, Nishimura S, Taniguchi T, Fujiu K, Manabe I, Nakamura K, Yamashita T, Saigusa R, Akamata K (2014). Simultaneous downregulation of KLF5 and Fli1 is a key feature underlying systemic sclerosis. Nat Commun..

[28] Rice LM, Padilla CM, McLaughlin SR, Mathes A, Ziemek J, Goummih S, Nakerakanti S, York M, Farina G, Whitfield ML (2015). Fresolimumab treatment decreases biomarkers and improves clinical symptoms in systemic sclerosis patients. J Clin Invest..

[29] Wahl SM, Hunt DA, Wakefield LM, McCartney-Francis N, Wahl LM, Roberts AB, Sporn MB (1987). Transforming growth factor type β induces monocyte chemotaxis and growth factor production. Proc Natl Acad Sci U S A..

[30] Arai M, Ikawa Y, Chujo S, Hamaguchi Y, Ishida W, Shirasaki F, Hasegawa M, Mukaida N, Fujimoto M, Takehara K (2013). Chemokine receptors CCR2 and CX3CR1 regulate skin fibrosis in the mouse model of cytokine-induced systemic sclerosis. J Dermatol Sci..

[31] Gerber EE, Gallo EM, Fontana SC, Davis EC, Wigley FM, Huso DL, Dietz HC (2013). Integrin-modulating therapy prevents fibrosis and autoimmunity in mouse models of scleroderma. Nature..

[32] Fleischmajer R, Perlish JS, Reeves JR (1977). Cellular infiltrates in scleroderma skin. Arthritis Rheum..

[33] Yoshizaki A, Iwata Y, Komura K, Ogawa F, Hara T, Muroi E, Takenaka M, Shimizu K, Hasegawa M, Fujimoto M (2008). CD19 regulates skin and lung fibrosis via Toll-like receptor signaling in a model of bleomycin-induced scleroderma. Am J Pathol..

[34] Yoshizaki A, Yanaba K, Yoshizaki A, Iwata Y, Komura K, Ogawa F, Takenaka M, Shimizu K, Asano Y, Hasegawa M (2010). Treatment with rapamycin prevents fibrosis in tight-skin and bleomycin-induced mouse models of systemic sclerosis. Arthritis Rheum..

[35] Katsumoto TR, Whitfield ML, Connolly MK (2011). The pathogenesis of systemic sclerosis. Annu Rev Pathol..

[36] Wynn TA, Barron L (2010). Macrophages: master regulators of inflammation and fibrosis. Semin Liver Dis..

[37] Maier C, Ramming A, Bergmann C, Weinkam R, Kittan N, Schett G, Distler JHW, Beyer C (2017). Inhibition of phosphodiesterase 4 (PDE4) reduces dermal fibrosis by interfering with the release of interleukin-6 from M2 macrophages. Ann Rheum Dis..

[38] Khanna D, Denton CP, Jahreis A, van Laar JM, Frech TM, Anderson ME, Baron M, Chung L, Fierlbeck G, Lakshminarayanan S (2016). Safety and efficacy of subcutaneous tocilizumab in adults with systemic sclerosis (faSScinate): a phase 2, randomised, controlled trial. Lancet..

[39] Tacke F, Randolph GJ (2006). Migratory fate and differentiation of blood monocyte subsets. Immunobiology..

[40] Carlin LM, Stamatiades EG, Auffray C, Hanna RN, Glover L, Vizcay-Barrena G, Hedrick CC, Cook HT, Diebold S, Geissmann F (2013). *Nr4a1*-dependent Ly6C^low^ monocytes monitor endothelial cells and orchestrate their disposal. Cell..

[41] Gibbons MA, MacKinnon AC, Ramachandran P, Dhaliwal K, Duffin R, Phythian-Adams AT, van Rooijen N, Haslett C, Howie SE, Simpson AJ (2011). Ly6Chi monocytes direct alternatively activated profibrotic macrophage regulation of lung fibrosis. Am J Respir Crit Care Med..

[42] Peng X, Zhang J, Xiao Z, Dong Y, Du J (2015). CX3CL1–CX3CR1 interaction increases the population of Ly6C^−^CX3CR1^hi^ macrophages contributing to unilateral ureteral obstruction-induced fibrosis. J Immunol..

[43] Lenzo JC, Turner AL, Cook AD, Vlahos R, Anderson GP, Reynolds EC, Hamilton JA (2012). Control of macrophage lineage populations by CSF-1 receptor and GM-CSF in homeostasis and inflammation. Immunol Cell Biol..

[44] Terry RL, Miller SD (2014). Molecular control of monocyte development. Cell Immunol..

[45] Zhang F, Wang H, Wang X, Jiang G, Liu H, Zhang G, Wang H, Fang R, Bu X, Cai S (2016). TGF-β induces M2-like macrophage polarization via SNAIL-mediated suppression of a pro-inflammatory phenotype. Oncotarget..

[46] Wang S, Meng XM, Ng YY, Ma FY, Zhou S, Zhang Y, Yang C, Huang XR, Xiao J, Wang YY (2016). TGF-β/Smad3 signalling regulates the transition of bone marrow-derived macrophages into myofibroblasts during tissue fibrosis. Oncotarget..

[47] Wang Y, Fan PS, Kahaleh B (2006). Association between enhanced type I collagen expression and epigenetic repression of the *FLI1* gene in scleroderma fibroblasts. Arthritis Rheum..

[48] Taniguchi T, Asano Y, Akamata K, Noda S, Takahashi T, Ichimura Y, Toyama T, Trojanowska M, Sato S (2015). Fibrosis, vascular activation, and immune abnormalities resembling systemic sclerosis in bleomycin-treated Fli-1-haploinsufficient mice. Arthritis Rheumatol..

[49] Takahashi T, Asano Y, Sugawara K, Yamashita T, Nakamura K, Saigusa R, Ichimura Y, Toyama T, Taniguchi T, Akamata K (2017). Epithelial Fli1 deficiency drives systemic autoimmunity and fibrosis: possible roles in scleroderma. J Exp Med..

[50] Distler JH, Distler O (2008). Criteria to select molecular targets for anti-fibrotic therapy. Rheumatology (Oxford)..

