# Supplementary figures and images for: Blockade of TGF-β/Smad signaling by the small compound HPH-15 ameliorates experimental skin fibrosis
Source: Arthritis Res Ther. 2018 Mar 15;20:46. doi: 10.1186/s13075-018-1534-y (PMC5855969; doi:10.1186/s13075-018-1534-y)

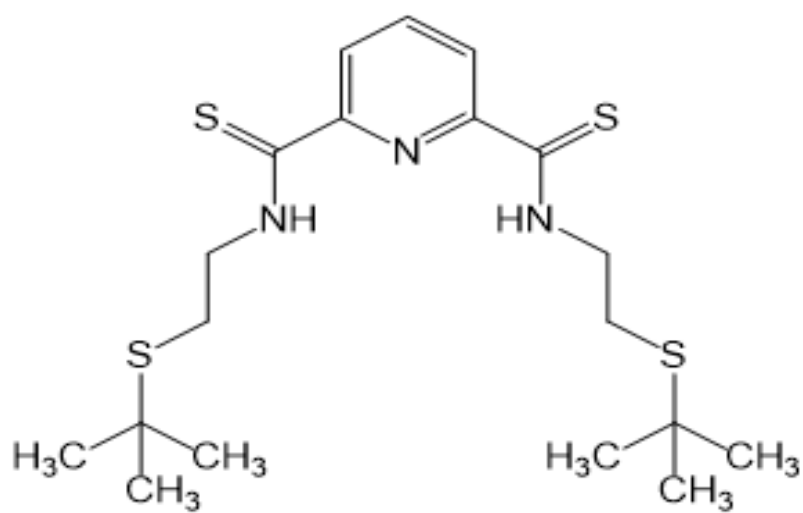

**Supplemental figure 1**  
**Luong VH, et al**

Supplement: Supplementary file 1 — Figure S1. The molecular structure of HPH-15. (PDF 7 kb) [file 13075_2018_1534_MOESM1_ESM.pdf]

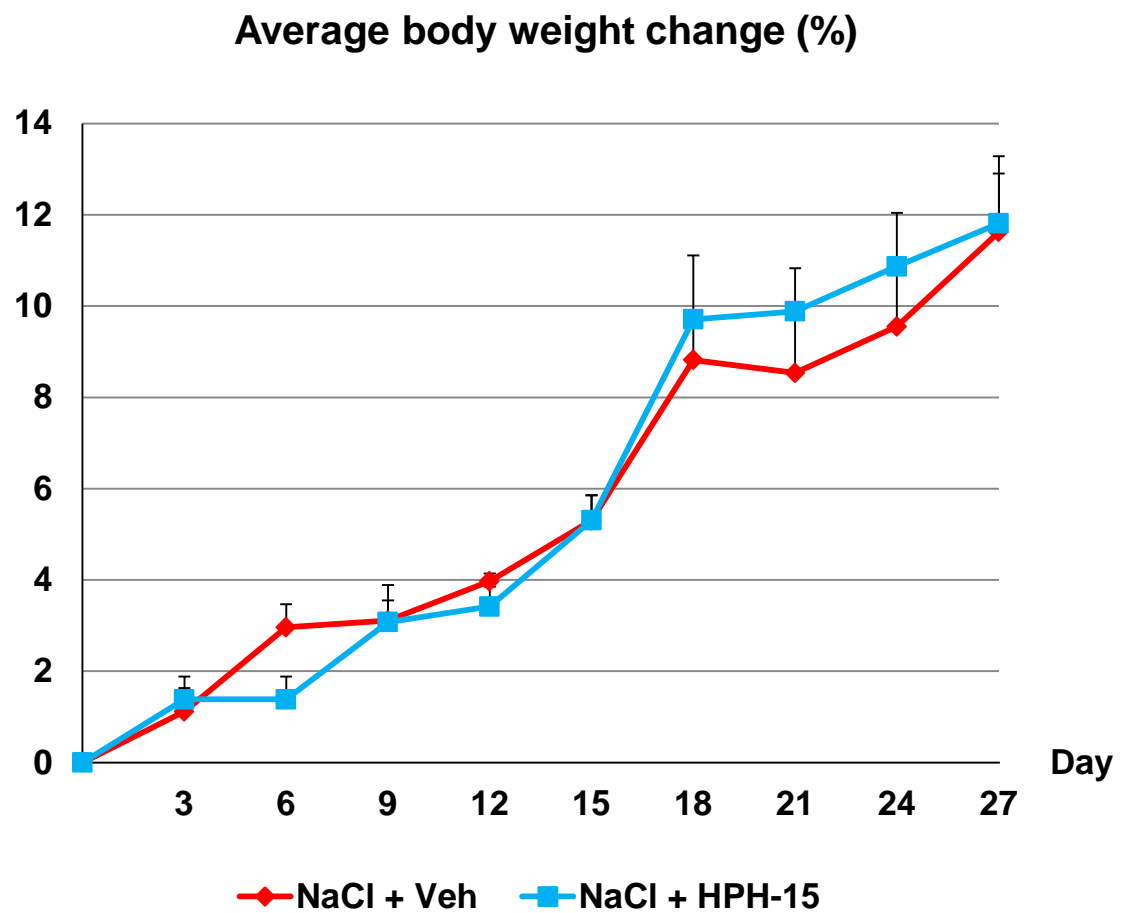

**Supplemental figure 2**  
**Luong VH, et al**

Supplement: Supplementary file 2 — Figure S2. HPH-15 treatment did not affect the growing of the mice. The body weight changes of mice treated with vehicle or HPH-15 were assessed every 3 days. All values represent mean ± SEM; n = 5 in each group. (PDF 6 kb) [file 13075_2018_1534_MOESM2_ESM.pdf]

**NaCl**

**BLM**

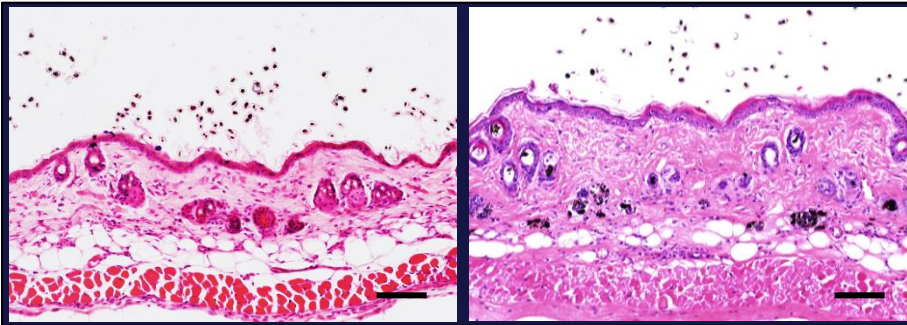

**Supplemental figure 3**  
**Luong VH, et al**

Supplement: Supplementary file 3 — Figure S3. Every-other-day injection of bleomycin for 2 weeks induced significant skin fibrosis. Back skin of NaCl- or bleomycin-injected mice was harvested on day 14 and stained with H&E. Scale bar = 100 μm. n = 3 in each group. (PDF 80 kb) [file 13075_2018_1534_MOESM3_ESM.pdf]

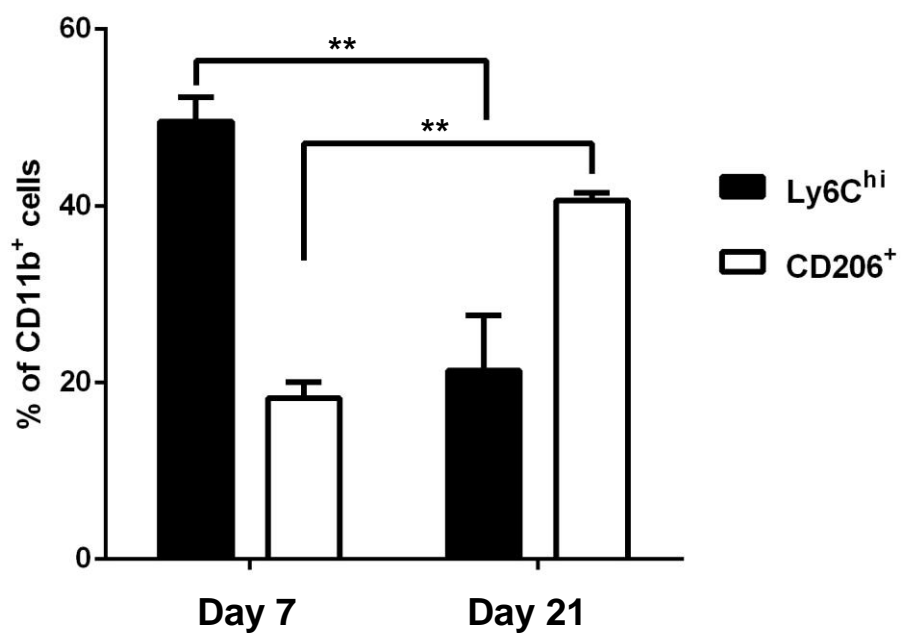

**Supplemental figure 4**  
**Luong VH, et al**

Supplement: Supplementary file 4 — Figure S4. The inverse proportional change of Ly6Chi macrophages and CD206+ M2 macrophages from the inflammation stage (day 7) to the fibrotic stage (day 21). The single-cell suspension obtained from the back skin of bleomycin-injected C57BL/6 mice on day 7 and day 21 was stained with the mAbs against CD45, CD11b, Ly6C, and CD206. Stained samples were analyzed using the FACSCanto II system. Data were analyzed using FlowJo software version 7. All values represent mean ± SEM. n = 3 at each time point. ** p ≤ 0.01. (PDF 49 kb) [file 13075_2018_1534_MOESM4_ESM.pdf]
